# Supplementary material for: Spatial pattern of COVID-19 deaths and infections in small areas of Brazil
Source: PLoS One. 2021 Feb 11;16(2):e0246808. doi: 10.1371/journal.pone.0246808 (PMC7877657; doi:10.1371/journal.pone.0246808)
Supplement: Attachment — Submitted filename: PONE-D-20-27148__PlosOne Brazil 9-2020 reviewer-1.docx [file pone.0246808.s001.docx]

PLOS ONE

**Spatial pattern of COVID-19 deaths and infections in small areas of Brazil**

--Manuscript Draft--

| **Manuscript Number:** | PONE-D-20-27148 |
| --- | --- |
| **Article Type:** | Research Article |
| **Full Title:** | Spatial pattern of COVID-19 deaths and infections in small areas of Brazil |
| **Short Title:** | Spatial pattern of COVID-19 deaths and infections in small areas of Brazil |
| **Corresponding Author:** | Emerson Augusto Baptista, Ph.D. Shanghai University  Shanghai, CHINA |
| **Keywords:** | COVID-19; deaths; infections; small-areas; spatial analysis; Brazil |
| **Abstract:** | Background: As at mid August, 2020, Brazil is the second most affected country by the COVID-19 pandemic, with large regional and social differences. In this study, using data from Brazilian Ministry of Health, we analyze the spatial patterns of infection and mortality from Covid-19 across small areas in Brazil.  Methods: we apply spatial autoregressive Bayesian models and estimate the risks of infection and mortality, taking into account age and sex composition of the population.We also perform a decomposition analysis to study how age composition impacts the differences in mortality and infection rates across regions.  Results: our results indicate that death and infections are spatially distributed forming clusters and hotspots especially in the Northern Amazon, Northeast coast and Southeast of the country.  Conclusion: the high mortality risks in the Southeast part of the country, which harbours the major cities can, be explained by the high proportion of elderly population. In the less developed areas of North and Northeast, there is a combination of high infection among young adults, socioeconomicand health access backwardness that results in more deaths. |
| **Order of Authors:** | Everton Emanuel Campos de Lima, Ph.D |
|  | Ezra Gayawan |
|  | Emerson A. Baptista |
|  | Bernardo Lanza Queiroz |
| **Additional Information:** |  |
| **Question** | **Response** |
| **Financial Disclosure**  Enter a financial disclosure statement that describes the sources of funding for the work included in this submission. Review the [submission guidelines](http://journals.plos.org/plosone/s/submission-guidelines#loc-financial-disclosure-statement) for detailed requirements. View published research articles from [*PLOS ONE*](http://journals.plos.org/plosone/) for specific examples.  This statement is required for submission and **will appear in the published article** if the submission is accepted. Please make sure it is accurate. | The author(s) received no specific funding for this work00 |

| **Unfunded studies**  Enter: *The author(s) received no specific funding for this work.*  **Funded studies**  Enter a statement with the following details:   - Initials of the authors who received each award - Grant numbers awarded to each author - The full name of each funder - URL of each funder website - Did the sponsors or funders play any role in the study design, data collection and analysis, decision to publish, or preparation of the manuscript? - **NO** - Include this sentence at the end of your statement: *The funders had no role in study design, data collection and analysis, decision to publish, or preparation of the manuscript.* - **YES** - Specify the role(s) played. |  |
| --- | --- |
| * typeset |  |
| **Competing Interests**  Use the instructions below to enter a competing interest statement for this submission. On behalf of all authors, disclose any [competing interests](http://journals.plos.org/plosone/s/competing-interests) that could be perceived to bias this work—acknowledging all financial support and any other relevant financial or non- financial competing interests.  This statement **will appear in the published article** if the submission is accepted. Please make sure it is accurate. View published research articles from [*PLOS ONE*](http://journals.plos.org/plosone/) for specific examples. | The authors have declared that no competing interests exist. |

| **NO authors have competing interests**  Enter: *The authors have declared that no competing interests exist.*  **Authors with competing interests**  Enter competing interest details beginning with this statement:  *I have read the journal's policy and the authors of this manuscript have the following competing interests: [insert competing interests here]* |  |
| --- | --- |
| * typeset |  |
| **Ethics Statement**  Enter an ethics statement for this submission. This statement is required if the study involved:   - Human participants - Human specimens or tissue - Vertebrate animals or cephalopods - Vertebrate embryos or tissues - Field research   Write "N/A" if the submission does not require an ethics statement.  General guidance is provided below. Consult the [submission guidelines](http://journals.plos.org/plosone/s/submission-guidelines#loc-guidelines-for-specific-study-types) for detailed instructions. **Make sure that all information entered here is included in the Methods section of the manuscript.** | Analysis of secondary data (individuals cannot be identified) |

| **Format for specific study types**  **Human Subject Research (involving human participants and/or tissue)**   - Give the name of the institutional review board or ethics committee that approved the study - Include the approval number and/or a statement indicating approval of this research - Indicate the form of consent obtained (written/oral) or the reason that consent was not obtained (e.g. the data were analyzed anonymously)   **Animal Research (involving vertebrate animals, embryos or tissues)**   - Provide the name of the Institutional Animal Care and Use Committee (IACUC) or other relevant ethics board that reviewed the study protocol, and indicate whether they approved this research or granted a formal waiver of ethical approval - Include an approval number if one was obtained - If the study involved *non-human primates*, add *additional details* about animal welfare and steps taken to ameliorate suffering - If anesthesia, euthanasia, or any kind of animal sacrifice is part of the study, include briefly which substances and/or methods were applied   **Field Research**  Include the following details if this study involves the collection of plant, animal, or other materials from a natural setting:   - Field permit number - Name of the institution or relevant body that granted permission |  |
| --- | --- |
|  |  |
| **Data Availability**  Authors are required to make all data underlying the findings described fully available, without restriction, and from the time of publication. PLOS allows rare exceptions to address legal and ethical concerns. See the [PLOS Data Policy](http://journals.plos.org/plosone/s/data-availability) and [FAQ](http://journals.plos.org/plosone/s/data-availability#loc-faqs-for-data-policy) for detailed information. | Yes - all data are fully available without restriction |

| A Data Availability Statement describing where the data can be found is required at submission. Your answers to this question constitute the Data Availability Statement and **will be published in the article**, if accepted. |  |
| --- | --- |
| **Important:** Stating ‘data available on request from the author’ is not sufficient. If your data are only available upon request, select ‘No’ for the first question and explain your exceptional situation in the text box. |  |
| Do the authors confirm that all data underlying the findings described in their manuscript are fully available without restriction? |  |
| **Describe where the data may be found in full sentences. If you are copying our sample text, replace any instances of XXX with the appropriate details.**   - If the data are **held or will be held in a public repository**, include URLs, accession numbers or DOIs. If this information will only be available after acceptance, indicate this by ticking the box below. For example: *All XXX files are available from the XXX database (accession number(s) XXX, XXX.*). - If the data are all contained **within the manuscript and/or Supporting Information files**, enter the following: *All relevant data are within the manuscript and its Supporting Information files.* - If neither of these applies but you are able to provide **details of access elsewhere**, with or without limitations, please do so. For example:   *Data cannot be shared publicly because of [XXX]. Data are available from the XXX Institutional Data Access / Ethics Committee (contact via XXX) for researchers who meet the criteria for access to confidential data.*  *The data underlying the results presented in the study are available from (include the name of the third party* | This data is public available at: https://opendatasus.saude.gov.br/dataset/casos- nacionais |

| *and contact information or URL).*   - This text is appropriate if the data are owned by a third party and authors do not have permission to share the data.   * typeset |  |
| --- | --- |
| Additional data availability information: | Tick here if the URLs/accession numbers/DOIs will be available only after acceptance of the manuscript for publication so that we can ensure their inclusion before publication. |

Main document	[Click here to](https://www.editorialmanager.com/pone/download.aspx?id=27295064&guid=66033637-3cb7-4b5d-a2d5-172b895afdcd&scheme=1)

[access/download;Manuscript;Manuscript_Plos_one.docx](https://www.editorialmanager.com/pone/download.aspx?id=27295064&guid=66033637-3cb7-4b5d-a2d5-172b895afdcd&scheme=1)

# 1 Spatial pattern of COVID-19 deaths and infections in small areas of Brazil

2

# 3 Authors order

4 **1. Everton E .C. Lima**. Assistant Professor at the College of Philosophy and Human Sciences
5 (IFCH) at University of Campinas (UNICAMP) and research scientist at the Population
6 Studies Center (NEPO), [everton.emanuel@gmail.com](mailto:everton.emanuel@gmail.com) and/or [evertone@unicamp.br](mailto:evertone@unicamp.br).
7 **2. Ezra Gayawan.** Senior Lecturer, Department of Statistics, Federal University of
8 Technology, Akure, Nigeria.[egayawan@futa.edu.ng.](mailto:egayawan@futa.edu.ng)
9 **3. Emerson A. Baptista.** Assistant Professor. Asian Demographic Research Institute (ADRI),
10 Shanghai University, Shanghai 200444, China. [emersonaug@gmail.com](mailto:emersonaug@gmail.com). *(Corresponding*
11 *author)*.
12 **4. Bernardo L. Queiroz.** Associate Professor. Department of Demography, Universidade
13 Federal de Minas Gerais. [lanza@cedeplar.ufmg.br.](mailto:lanza@cedeplar.ufmg.br)

# 14 Abstract

15 As at mid August, 2020, Brazil is the second most affected country by the COVID-19
16 pandemic, with large regional and social differences. In this study, using data from Brazilian
17 Ministry of Health, we analyze the spatial patterns of infection and mortality from Covid-19
18 across small areas in Brazil. We apply spatial autoregressive Bayesian models and estimate
19 the risks of infection and mortality, taking into account age and sex composition of the
20 population. We also perform a decomposition analysis to study how age composition impacts
21 the differences in mortality and infection rates across regions. Our results indicate that death
22 and infections are spatially distributed forming clusters and hotspots especially in the
23 Northern Amazon, Northeast coast and Southeast of the country. The high mortality risks in
24 the Southeast part of the country, which harbours the major cities can be explained by the
25 high proportion of elderly in the population. In the less developed areas of the North and Northeast,
26 there is a combination of high infection among young adults, socioeconomic and those without access to health care
27 resulting in more deaths.

# 28 Introduction

29 The COVID-19 pandemic has proved to be a challenge that is capable of impacting public
30 health of all countries in the world with outcomes hitherto unimaginable. However, for
31 several reasons, such as political and economic, some countries, like Brazil, have faced
32 additional difficulties in controlling the pandemic. Brazil has become the new epicenter of this
33 disease^1^ as a result of actions of many local authorities who disregard WHO recommendations
34 and ignore strong measures of population isolation, mask wearing, testing and contact tracing^2-4^.
35 Brazil is characterized by large regional and socioeconomic disparities and high levels
36 of inequality in access to health services. Thus, one may expect these to impact on the
37 country’s number of infections and deaths caused by COVID-19 in different levels across
38 regions of the country. Additionally, at the onset of the pandemic, there was a positive
39 correlation between deaths and infections rates in a few cities in the southern part of the
40 country^5^, but recently, this disease has spread through less developed areas of the country^6-9^.
41 Besides health disparities, there are three components that need to be considered when
42 studying the pattern of COVID-19 in Brazil. First, the mortality rates are marked by a
43 considerable age gradient, that is, the number of deaths is significantly higher among the
44 elderly and, consequently, the population age structure is an important element to
45 understand the spread and the risk of mortality across areas^10-12^. Second, the number of deaths
46 is significantly higher among males than females^13^. We hypothesized that since women give
47 more attention to issues affecting their health, this could have a positive impact on mortality
48 risks. In addition, women are the majority among informal workers, domestic workers and the
49 unemployed^14^, that is, they are also more directly affected by the lockdown imposed in many
50 places in the country. Finally, as is the case elsewhere, pre-existent health problems or
51 comorbidities seem to increase mortality risks in the population^12^^,15^. Some studies have shown
52 that mortality is significantly higher among population strata that present any history of
53 cardiovascular	diseases,	diabetes or obesity^12^^,16^^,17^. The combination of these three
54 components, age structure, comorbidities and sex, may also play a role in the final number of
55 deaths.
56 In this paper, we used publicly available data from the Brazilian Ministry of Health to
57 estimate infection and mortality rates from COVID-19 in small-areas, taking into
58 consideration the population age structure and sex distribution, and the level of income
59 inequality across regions. We used a Bayesian hierarchical model for the 558 micro-regions of
60 Brazil, based on data available at the end of July. Estimates at the local level are very
61 important to develop proper public health interventions and evaluate the impact of the
62 pandemic. An important caveat is that we used information on cumulative infections and
63 death counts on a specific date. We did not control for the period in which the pandemic
64 started in each area of the country. Notwithstanding, we observed that areas that started the
65 pandemic in later stages also reported a significant increase in the pandemic and they are rapidly
66 catching-up in the number of cases and mortality relative to the first areas of the pandemic.

67

# 68 Methods

69 *Data source and level of analysis*
70 We use public available data from the Ministry of Health of Brazil, DATASUS, available at
71 [https://opendatasus.saude.gov.br/dataset/casos-nacionais^18^](https://opendatasus.saude.gov.br/dataset/casos-nacionais). Since 2009, there has been a
72 system of surveillance for Serious Acute Respiratory Syndrome (SARS) that collects
73 information about influenza and other respiratory illnesses across the country. The system
74 incorporates recent information on COVID-19 deaths and infections at municipal level, the
75 smallest geographical aggregation of the country.
76 Due to instability in the numbers of cases and exposed population, we aggregated the
77 municipality information to 558 microregions, as defined by the Brazilian National Statistical
78 Office (IBGE). These geographical areas are aggregated according to regional and
79 socioeconomic similarities, which provide a good benchmark to compare spatial distribution
80 of COVID-19 across the country.
81 We collected the information on July 20th, when Brazil had registered 77,226 deaths
82 in this database, and a total of 1,795,054 infections. From these numbers, the amount of
83 deaths was 45,013 for males and 32,213 for females – a male/female ratio of 1.39. For
84 infections, there is a different picture, a male/female ratio of 0.89, with 846,744 and 948,310
85 infections for males and females, respectively.

86

87 *Statistical method*
88 We used a Bayesian formulation of a spatial autoregressive model (SAM), suitable for
89 smoothing and stabilizing spatial estimates, to study the spatial pattern of the infections and
90 mortality^19^.
91 The observed count of cases 𝑌_𝑖_ in area *i* is modelled using a Poisson distribution with
92 Mean 𝐸_𝑖_𝜃_𝑖_, where 𝐸_𝑖_ is the expected count of cases and 𝜃_𝑖_ is the relative risk in area *i*. The
93 relative risk (𝜃_𝑖_) quantifies whether an area has a higher (𝜃_𝑖_ > *1*) or lower (𝜃_𝑖_ < *1*) risk than
94 the average risk in the standard population. In the case of mortality, because of the large
95 number of zeros recorded in most of the study areas, a zero-inflated Poisson model was
96 considered appropriate. If 𝑌_𝑖_~𝑃𝑜(𝐸_𝑖_𝜃_𝑖_), *i* = 1, ...*n*, then the relative risk (𝜃_𝑖_) can be modelled
97 through a log link function such that

98	𝑙𝑜𝑔(𝜃_𝑖_) = 𝛼 + 𝑢_𝑖_ + 𝑣_𝑖_ + 𝛾_𝑖_	(1)

99	Where *α* is the overall risk in the study areas, (𝑢_𝑖_ + 𝑣_𝑖_) is an area random effect, such

100	that 𝑢_𝑖_ models the spatial dependency between the relative risks and 𝑣_𝑖_ is an unstructured

101

exchangeable component that model uncorrelated noise, such that 𝑣_𝑖_~𝑁(0, 𝜎^2^), while 𝛾

is a

102 function assumed for the age categories. The structured spatial component was considered

𝑣

𝑖

103 through the conditional autoregressive (CAR) distribution, which smooth the risks according
104 to some neighbourhood structure that considers two areas as neighbours if they share a
105 common boundary. The prior is specified as:
106 106

2

(𝑢_𝑖_|𝑢_−𝑖_)~𝑁 (𝑢̅_𝛿_ , ^𝑢^ ),

𝛿

𝑖	𝑛𝛿𝑖

107 107

Where𝑢̅_𝛿_	= 𝑛^−1^ ∑_𝑗∈𝛿_ 𝑢_𝑗_, 𝛿_𝑖_represent the set of neighbours and𝑛_𝛿_

is the number of

𝑖	𝛿𝑖	𝑖	𝑖

108 neighbours of area*i*. The unstructured component 𝑣_𝑖_ was considered as independent and
109 109

identically distributed normal with zero mean and variance𝛿^2^. The function 𝛾

was modelled

𝑣	𝑖

110 through a random walk of order 2 and also considered as linear effect and modelled together
111 with the intercept using Gaussian priors with mean 0 and precision 0.001.
112 The Bayesian inference was determined using the integrated nested Laplace
113 approximation (INLA)^20^, as implemented in the R-INLA package^21^. For each of cases and
114 mortality we considered four models for each gender by varying the forms and terms in model
115 (1) and compared model performance through deviance information criterion (DIC) and
116 Watanabe information criterion (WAIC), where the model with the lowest value for each of the
117 criterion is considered the best. Results reported are based on those of the best model.
118 118
119 *Decomposition of mortality and infections risks according to age structure*
120 In addition to the spatial analysis, we performed a decomposition analysis to investigate the
121 possible effects of population age structure on mortality and infections. Thus, we sought to
122 analyze the contribution of this factor on possible regional differences in the risks of death and
123 infection across the regions^22^.
124 We decomposed each difference in mortality and infection risks between two regions
125 based on Kitagawa ^23^ formulation:
126 126
127 127

𝑅*2* − 𝑅*1* = ∑𝑛 (𝑃*2*𝑖 − P*1*𝑖) × (𝑅*2*𝑖−𝑅*1*𝑖))+∑𝑛

(𝑅*2*𝑖 − 𝑅*1*𝑖) × (𝑃*2*𝑖−𝑃*1*𝑖))

(2)

𝑖=*1*

*_2_*	𝑖=*1	_2_*

128 Where, *R2* and *R1* are the overall risks of an outcome in populations 2 and 1, and *i* is
129 the category of predictor variable, age. *P2i* and *P1i* are the proportion of population 2 and 1 in
130 category i of the predictor, and *R2i* and *R1i* are the risks of outcome in population 2 and 1 in
131 category i of the predictor.
132 132

# 133 Results

134 Figure 1 presents the box plot of the posterior means of the relative risks of morality by age
135 group from all the microregions. Mortality risks from COVID-19 were low at age group 0-4
136 years but increased steadily with age. Though, on the average, the relative risks for all the
137 microregions are below one unit across the age groups, the figure reveals that from age
138 group 40-44 years on, there are several regions standing as outliers, where the risks are above
139 one, and this is more pronounced for individuals aged 80 years and above. These patterns of
140 risks were observed for males and females, but mortality risks for males are greater than for
141 females across all ages.

# 142 Figure 1: Relative mortality risk according to conventional five years age groups in

143 **Brazil, 2020.**
144 [Figure 1 about here]
145 Source: Brazilian Ministry of Health 2020.
146 146
147 Figure 2 and Figure 3 show the spatial distribution of the posterior means for relative
148 risks for mortality and infection from COVID-19, respectively. The findings from the spatial
149 analysis are not reported in the conventional 5-year age groups, rather they are presented in
150 three large age groups (0-39, 40-59, and >60 years). We adopt these age ranges because our
151 estimates show that the likelihood of deaths is quite small under the 40 years of age, but
152 increases slowly up to age 60 and then rise exponentially. Additionally
153 the age pattern of the relative risks of infection follows a different structure, it peaks between
154 the ages of 20-54 years old, similar to the findings of another study^13^ (data not shown),. Thus, to use the same
155 scale of comparison between deaths and infection cases, we opted to present all spatial analyses
156 of these age groups in both cases.
157 In general, the results show that the relative risks for mortality (Figure 2) by
158 microregion and sex are remarkably higher for older adults. Similar spatial pattern is observed
159 for males and for females. There are few distinctions about the geographical distribution of
160 the risks of deaths between males and females across the age groups. From children to young-
161 adults, the relative risks almost do not surpass the value of 0.20 across the microregions.
162 Between the ages of 40 and 59 years old, there were isolated spots of high mortality risk, but
163 in few places around the North Amazon Metropolitan area (Manaus) and in Northeast coast
164 microregion of Fortaleza (Ceará). These two areas have been heavily affected in terms of
165 cases and deaths in the first months of the pandemic^7,^ ^24^.

# 166 Figure 2: Spatial pattern of COVID-19 mortality risks by gender and microregions of

167 **Brazil, 2020.**
168 [Figure 1 about here]
169 Source: Brazilian Ministry of Health 2020.
170 Additionally, in the Rio de Janeiro and São Paulo microregions, located in the
171 Southeast of Brazil, there are spatial clusters of high mortality risks among this age
172 group. This could be expected because, being business and touristic areas, these two locations
173 were the first to report cases of SARS-CoV-2 infection in the country^7,^ ^24^. Once more, it is
174 important that we take into consideration the time that the pandemic hit a certain location,
175 which may affect its relative risks in comparison with other locations. Although, that fact does
176 not entirely compromise our estimates, because we also see many other areas in the country
177 that registered much later cases of COVID-19 and they are showing relative mortality risks
178 similar to the avant-garde microregions.
179 For the population above 60 years of age, the results indicate additional spatial
180 clusters. There exists a cluster of higher mortality risks that extends from the Southeast part of
181 the country and connects to the middle of the country. In addition, North Amazon and the
182 coast of the Northeast regions present a high risk of mortality for the elderly due to COVID-19.
183 One interesting fact is that the highest mortality risks are basically located in Northern and
184 Northeastern coast areas of Brazil, despite the South and Southeast microregions presenting
185 older population age structures ^25^, and one would expect that in these last regions overall
186 mortality due to COVID-19 might be higher, but this is not the case, especially in the aged
187 South region of the country. We may speculate that socioeconomic disparities and worse
188 conditions for access health facilities in Northern parts of Brazil play a role to increase
189 mortality risks among elderly groups in these areas.

# 190 Figure 3: Spatial pattern of COVID-19 infected risks by sexes and microregions of

191 **Brazil, 2020.**
192 [Figure 3 about here]
193 Source: Brazilian Ministry of Health 2020.
194 A second important finding is that the spatial pattern of infection risks is also highly
195 concentrated in the North and Northeast of Brazil (Figure 3). This time, we see a significant
196 contribution of the young-adult population to increase the infection risks in these areas, while
197 in other parts of the country the risks of COVID-19 cases are much lower. Perhaps, besides
198 socioeconomic, the high infection risks among the young and adult population could explain
199 the higher risks of mortality in these areas as well.
200 We now turn our analysis to the decomposition effects. Figure 4 shows that
201 decomposing the effects of mortality and infection risks in terms of differences in age
202 compositions and other effects, captured by differences in risks. To facilitate the analyses, we
203 grouped the microregions according to macroregions and selected the region with the highest proporportion in the oldest age group (the South), as a benchmark of comparison with the others. This way, we could analyze how
204 the age structure affects mortality and infections by Coronavirus disease across the country.

# 205 Figure 4: Differences in mortality and infection risks decomposed by age composition

206 **and other effects, comparison between macroregions of Brazil, South versus other**

# 207 regions.

208 [Figure 4 about here]
209 Source: Brazilian Ministry of Health 2020.
210 The decomposition exercise did not show major differences among females and
211 males, hence, we describe the findings for both sexes. In terms of mortality risks, with
212 exception of comparison with Midwest, the South region has lower risk mortality than other
213 regions. Age composition tends to reduce these differences, especially relative to the younger
214 aged Northern and Northeastern regions, but other elements are responsible for increasing the
215 differences in mortality risks between the South and other locations of Brazil.
216 When we looked at infection risks, age composition played a small role in the differences
217 between macroregions, all areas presented a higher risk infection of COVID-19 than the South,
218 and many unobserved factors are responsible for these regional differences in the number of
219 cases. This last finding does not exclude the fact that age structure also played an important role
220 in the spatial pattern of infection, as we could see in the previous estimates disaggregated by
221 microregions.
222 223

# 223 Discussion

224 In most countries, we do not have only one COVID-19 outbreak going on. There are several
225 differences across regions in the rhythm and stage of the outbreaks^26^. Thus, it is very relevant
226 to investigate the patterns in infections and mortality across different regions of a
227 socioeconomically diverse country like Brazil^27,^ ^28^. One important issue is the limitation
228 regarding COVID-19 data in the country due to the lack of adequate testing and reporting. In this study, we
229 use a Bayesian hierarchical model to estimate rates of infection and mortality from COVID-19
230 based on the microregions of Brazil, taking into account the population age structure and sex
231 distribution, as well as the levels of socioeconomic inequality commonly expressed in
232 regional diversity.
233 Overall, our results suggest a different spatial pattern between the risks of death and
234 the relative risks of infection for both sexes. While the relative mortality risks by microregion
235 and sex are remarkably higher for older adults, particularly for men, the relative risks for
236 infections are concentrated among young adults, being proportionally higher among women.
237 But mortality risks are much greater for males than females.
238 We find that almost all the regions in the North and a good part of the microregions in
239 the Northeast coast appear to be at a disadvantage in terms of the overall risk of mortality,
240 when compared to the Southeast and South regions of the country. These are important points
241 to be considered coping with COVID-19, a disease that is more severe in the older population.
242 Even with a younger demograhic, many locations in the North and Northeast have higher
243 overall mortality rate, which could be due to overall worse health conditions. In addition,
244 these areas also present a great number of infections, in spite of the protective factor of a
245 more youthful population, the microregions of the North and Northeast of the country have higher
246 mortality rates due to age for certain causes of death that can accentuate the risks of
247 complications by COVID-19^29,^ ^30^.
248 For instance, Baptista and Queiroz^31^ observed a rapid increase in C__________ D_____(CVD) mortality from
249 2001 to 2015 in the Northern and Northeast parts of the countries, whereas in the South and
250 Southeast they found a steady decline. Overall, they also show that in approximately 75% of
251 Brazilian microregions, men have higher mortality rates than women. The Northern and
252 Northeastern regions are the least developed socioeconomically regions of the country and
253 have higher mortality rates than the others. França, Passos^30^ also show that mortality rates and
254 prevalence of diabetes are concentrated in this region of the country^25,^ ^32^.
255 This scenario, combined with the weakening of measures to deal with the pandemic,
256 such as those indicated by the WHO, are elements that need to be considered in mitigating
257 possible future short and medium term scenarios for the regional impacts of this pandemic.
258 Areas that, even before the pandemic, already presented greater risks of general mortality, did
259 not have a good health infrastructure, a large part of the population needs emergency
260 assistance from the government, especially elderly people with
261 comorbiditiesand who are not adhering to the measures of social isolations, especially at the moment of
262 the greatest spread of the pandemic, are exposed to greater risks of collapse in the health
263 system and can present a considerable increase in the number of deaths by COVID-19^9^.
264 265

# 265 Acknowledgements

266 Gayawan contribution was funded by the São Paulo Research Foundation (FAPESP) [grant
267 2018/18649-7] and Lima and Gayawan thank the University of Campinas Research Fund -
268 FAEPEX for support.

# 269 References

270 1. Dong E, Du H, Gardner L. An interactive web-based dashboard to track COVID-19 in real
271 time. *The Lancet Infectious Diseases* 2020; **20**: 533-4.
272 273
273 2. The Lancet. COVID-19 in Brazil: "So what?". *The Lancet* 2020; **395**: 1461.
274 275
275 3. Jorge DC, Rodrigues MS, Silva MS, et al. Assessing the nationwide impact of COVID-19
276 mitigation policies on the transmission rate of SARS-CoV-2 in Brazil. *medRxiv* 2020.
277 278
278 4. Dantas RCC, Campos PA, Rossi I, Ribas RM. Implications of social distancing in Brazil in
279 the COVID-19 pandemic. *Infection Control & Hospital Epidemiology* 2020.
280 281
281 5. Souza CDFd, Paiva JPSd, Leal TC, Silva LFd, Santos LG. Evolução espaçotemporal da
282 letalidade por COVID-19 no Brasil, 2020. *Jornal Brasileiro de Pneumologia* 2020; **46**:

284	e20200208.

285

286 6. Candido DS, Claro IM, Jesus JG, et al. Evolution and epidemic spread of SARS-CoV-2 in
287 Brazil. *Science* 2020; **eabd2161**.
288 288
289 7. Lima E, Vilela E, Peralta A, et al. Exploring excess of deaths in the context of covid
290 pandemic in selected countries of Latin America. *OSF Preprints* 2020.
291 291
292 8. França EB, Ishitani LH, Teixeira RA, et al. Óbitos por COVID-19 no Brasil: quantos e
293 quais estamos identificando? *Revista Brasileira de Epidemiologia* 2020; **23**: e200053.
294 294
295 9. Codeço CT, Villela D, Coelho F, et al. Estimativa de risco de espalhamento da COVID-19
296 no Brasil e o impacto no sistema de saúde e população por microrregião. 2020.
297 297
298 10. Dowd JB, Andriano L, Brazel DM, et al. Demographic science aids in understanding the
299 spread and fatality rates of COVID-19. *Proceedings of the National Academy of Sciences*

300	2020; **117**: 9696-8.

301

302 11. Goldstein JR, Lee RD. Demographic perspectives on the mortality of COVID-19 and
303 other epidemics. *Proceedings of the National Academy of Sciences* 2020.
304 304
305 12. Nepomuceno MR, Acosta E, Alburez-Gutierrez D, Aburto JM, Gagnon A, Turra CM.
306 Besides population age structure, health and other demographic factors can contribute to
307 understanding the COVID-19 burden. *Proceedings of the National Academy of Sciences* 2020;

308	**117**: 13881-3.

309

310	13. Baptista EA, Queiroz BL. Regional Covid-19 Mortality in Brazil by Age. *SocArXiv* 2020.

311

312 14. Barbosa ALNdH, Costa JS, Hecksher M. *Mercado de trabalho e pandemia da Covid-19 :*
313 *ampliação de desigualdades já existentes?* 2020 [cited 2020 21 August]; Available from:
314 <http://repositorio.ipea.gov.br/handle/11058/10186>
315 315
316 15. Wang B, Li R, Lu Z, Huang Y. Does comorbidity increase the risk of patients with
317 COVID-19: evidence from meta-analysis. *Aging* 2020; **12**: 6049–57.
318 318
319 16. Jordan RE, P A. Who is most likely to be infected with SARS-CoV-2? *The Lancet*
320 *Infectious Diseases* 2020; **20**: p995-6.
321 321
322 17. Shuchman M. Low- and middle-income countries face up to COVID-19. *Nature Medicine*

323	2020; **26**.

324

325 18. Ministry of Health. *Sistema de Informações sobre Mortalidade (SIM)*. 2020 [cited 2020
326 23 July]; Available from: https://opendatasus.saude.gov.br/dataset/casos-nacionais
327 327
328 19. LeSage JP. Bayesian estimation of spatial autoregressive models. *International Regional*

329	*Science Review* 1997; **20**: 113-29.

330

331 20. Rue H, Martino S, Chopin N. Approximate Bayesian inference for latent Gaussian models
332 by using integrated nested Laplace approximations. *Journal of the Royal Statistical Society:*
333 *Series B (Statistical Methodology)* 2009; **71**: 319-92.
334 334
335 21. Lindgren F, Rue H. Bayesian spatial modelling with R-INLA. *Journal of Statistical*

336	*Software* 2015; **63**: 1-25.

337

338 22. Medfod A, Trias-Llimos S. Population age structure only partially explains the large
339 number of COVID-19 deaths at the oldest ages. *Demographic Research* 2020; **43**: 533-44.
340 340
341 23. Kitagawa EM. Components of a difference between two rates. *Journal of the American*
342 *Statistical Association* 1955; **50**: 1168-94.
343 343
344 24. Pedro S. Peixoto, Diego Marcondes, Cláudia Peixoto, Oliva SM. Modeling future spread
345 of infections via mobile geolocation data and population dynamics. An application to
346 COVID-19 in Brazil. *PLoS ONE* 2020; **15**: e0235732.
347 347
348 25. Queiroz BL, Freire FHM, Lima EEC, Gonzaga MR. *O risco de mortalidade por*
349 *microrregiões e o efeito da composição etária no contexto da Covid-19*.	2020	[cited;
350 Available	from:	https://demografiaufrn.net/2020/05/04/o-risco-de-mortalidade-por-
351 microrregioes-e-o-efeito-da-composicao-etaria-no-contexto-da-covid-19/
352 352
353 26. Franch-Pardo I, Napoletano BM, Rosete-Verges F, Billa L. Spatial analysis and GIS in the
354 study of COVID-19. A review. *Science of The Total Environment* 2020; **739**: 140033.
355 355
356 27. Mollalo A, Vahedi B, Rivera KM. GIS-based spatial modeling of COVID-19 incidence
357 rate in the continental United States. *Science of The Total Environment* 2020; **728**: 138884.
358 358
359 28. Martines MR, Ferreira RV, Toppa RH, Assuncao L, Desjardins MR, Delmelle EM.
360 Detecting space-time clusters of COVID-19 in Brazil: mortality, inequality, socioeconomic
361 vulnerability, and the relative risk of the disease in Brazilian municipalities. *medRxiv* 2020.
362 362
363 29. Borges GM. Health transition in Brazil: regional variations and divergence/convergence in
364 mortality. *Cadernos de Saúde Pública* 2017; **33**: e00080316.
365 365
366 30. França EB, Passos VMdA, Malta DC, et al. Cause-specific mortality for 249 causes in
367 Brazil and states during 1990–2015: a systematic analysis for the global burden of disease
368 study 2015. *Population Health Metrics* 2017; **15**.
369 369
370 31. Baptista EA, Queiroz BL. The relation between cardiovascular mortality and
371 development. *Demographic Research* 2019; **41**: 1437-52.
372 372
373 32. Queiroz BL, Freire F, Lima E, Gonzaga MR, Baptista EA. Patterns of Geographic
374 Variation of Mortality by Causes of Death for Small Areas in Brazil, 2010. In: Jivetti B,
375 Hoque MN, editors. *Population Change and Public Policy*; 2020.

Figure 1	[Click here to access/download;Figure;Figure 1.tif](https://www.editorialmanager.com/pone/download.aspx?id=27295074&guid=ad1119df-cd0e-4f6d-9724-d0737e5cf071&scheme=1)

Figure 2	[Click here to access/download;Figure;Figure 2.tiff](https://www.editorialmanager.com/pone/download.aspx?id=27295075&guid=834a0689-f5a3-47cd-aa46-355e316e10c9&scheme=1)

Figure 3	[Click here to access/download;Figure;Figure 3.tiff](https://www.editorialmanager.com/pone/download.aspx?id=27295076&guid=98ab5b3e-f7dc-40a2-8c0d-a42433222f64&scheme=1)

Figure 4	[Click here to access/download;Figure;Figure 4.tiff](https://www.editorialmanager.com/pone/download.aspx?id=27295077&guid=d16dd8e4-99bc-42be-80fc-bef6a2a97663&scheme=1)
