# Supplementary material for: Spatial pattern of COVID-19 deaths and infections in small areas of Brazil
Source: PLoS One. 2021 Feb 11;16(2):e0246808. doi: 10.1371/journal.pone.0246808 (PMC7877657; doi:10.1371/journal.pone.0246808)
Supplement: Attachment — Submitted filename: PONE-D-20-27148R1 r2r.docx [file pone.0246808.s002.docx]

**LETTER TO REVIEWERS**

**To: Editor(s) of PLOS ONE**

**From: Emerson Baptista**

**Date: December, 07 2020**

**Subject**: Re-submission of manuscript “*Spatial pattern of Covid-19 deaths and infections in small areas of Brazil*”.

Dear editors,

We are submitting the revised version of the original article “Spatial pattern of Covid-19 deaths and infections in small areas of Brazil”, and our detailed response to the reviewers. We hope that the paper will be of interest to *Plos One* readers and, if required, we are ready to make more changes or amendments that would improve it further and make it suitable for publication in the journal.

With best wishes

Everton E. C. de Lima, Ezra Gayawan, Emerson Baptista and Bernardo Queiroz

*Thank you for carefully reading our paper and for providing many thoughtful comments. We believe that this revised version has been significantly improved as a result. Changes in the original text are in* ***RED****.*

**General comments for editorial board**

Concerning the permission from the original copyright holder of the maps, Figure(s) [2 and 3] to publish the content specifically under the CC BY 4.0 license. This is not necessary, because the maps are drawn using shapefiles, public available by the National Statistical Office (IBGE) and also accessible by the R package geobr, <https://cran.r-project.org/web/packages/geobr/index.html>.

We have edited the manuscript to meet the PLOS ONE's style requirements. In addition, we have also thoroughly copyedit manuscript for language usage, spelling, and grammar by a native speaker.

Here we list all the comments and detail our response and the corresponding revisions in the paper. *Changes in the original text are in* ***RED****.*

**Reviewer #1**:

The paper describes the geographic distribution of COVID-19 throughout Brazil, attributing non-homogeneity to demographics, specifically age.

Specific comments and edits to conform to standard North American English are included in the attached document.

**We hypothesized that since women give more attention to issues affecting their health, this could have a positive impact on mortality risks. 1) Or could this be associated with being in the home as opposed to outside of the home (related to employment opportunities for men vs. women in Brazil)?**

*Response: Research from European countries suggests that women see Covid-19 as a serious health issue and risk. Also, in Brazil most women in the labor market are in the informal sector, thus they also would be prone to have to leave the house to work.*

*Galasso, Vincenzo, et al. Gender differences in COVID-19 related attitudes and behavior: Evidence from a panel survey in eight OECD countries. No. w27359. National Bureau of Economic Research, 2020.*

***2) SARS CoV-1 and SARS CoV-2 are not influence viruses. Therefore, it is not clear what this surveillance system is actually tracking. Perhaps a sentence that says: The Brazilian respiratory disease surveillance system tracks influence, SARS and COVID-19 illnesses.***

*Response: We have made changes in the manuscript, accordingly, to clarify this issue, as stated by the reviewer.*

3) Define municipal level.

*Response: We have precise the definition of municipality in the manuscript.*

**Reviewer #2:**

**1) The manuscript presents interesting findings. But it needs to include essential confounding variables in the spatial analysis. Regarding mortality, the essential variable in this spatial analysis is the access to intensive care beds. This may explain part of the higher mortality in the north and northeast, which has the lowest number of beds per 100k of the country. It is important in this spatial analysis to incorporate this variable, perhaps it is more important than the distribution by sex and age in these regions. Another variable that can impact mortality is the coverage of primary care and coverage of supplementary private health.**

*Response: Brazilian Health Care System – we have reanalyzed the data and include number of intensive care units and number of physicians and the results have been presented. We also include a substantive discussion based on recent research in Brazil related to the Covid-19 pandemic. For example, Noronha et al (2020) show that the number of health care beds in Brazil is about half of what is observed in OECD countries. Moreover, the spatial distribution is very unequal across the country. According to their analysis, the regions with lowest supply are concentrated in Amazonas, Pará, Roraima, Minas Gerais and Ceará. The supply of ICU is also very unequal across the country, with most of regions in the North and Northeast showing lowest supply of tertiary care and highly concentrated in the state capitals. Finally, Noronha et.al (2020) discuss the distance from each city to a main health care hub and show that regions in the North and Northeast have the longest distance to a city with adequate offer of ICU beds. Also, Pereira et al (2020) show that a large variation in access to health care in Brazil. The access to Intensive Care Units varies across cities and regions and is much lower in poorer areas.*

*Noronha, Kenya Valeria Micaela de Souza, et al. "The COVID-19 pandemic in Brazil: analysis of supply and demand of hospital and ICU beds and mechanical ventilators under different scenarios."* ***Cadernos de Saúde Pública*** 36 (2020): e00115320.

*Pereira, Rafael HM, et al. "Geographic access to COVID-19 healthcare in Brazil using a balanced float catchment area approach."* ***medRxiv*** *(2020).*

*Oliveira, Ana Paula Cavalcante de et al. "Challenges for ensuring availability and accessibility to health care services under Brazil’s Unified Health System (SUS)."* ***Ciência & Saúde Coletiva*** *22 (2017): 1165-1180.*

**2) In addition, the analysis has to adjust the 558 micro-regions for the moment of the first case and count the same first days for the different regions. Only in this way is it possible to verify regional differences in terms of mortality and infection risks.**

*Response: on the count of the first day of occurrence for each municipalities, since we used the cumulative count of cases and deaths, it is assumed that other municipalities have zero counts as of the date the first cases was recorded in the country. This explains why, in the case of mortality, we used a zero-inflated Poisson model, because no death was recorded in many of the municipalities as at the time of the study and it would make no sense to exclude them for that reason. Thus, our model appropriately account for zero cases for the periods when the events (infection or death) were yet to occur.*

**Reviewer #3**:

**1) I recommend to make (indirect) standardization by age group in the different region to support the analysis comparing the microregions among the Brazilian regions, apparently they may be so different (example: North (Amazon) Region with South West region).**

*Response: We thank the review for this comment. Population age structure across regions in Brazil is indeed quite different. Population age structure is younger in the North and Northeast and older in the South and Southeast. In 2018, about 10% of the Brazilian population was over 65 years of age, but the variation between Federation Units can range from 13% in Rio Grande do Sul to 7% in Mato Grosso. This means that we cannot directly compare mortality levels from one state to another because it can show the impact of both mortality levels and age structure. We opt to standardize across all regions so we can compare the whole country to a standard. In the model, we incorporate the differentials in population age structure, so we are showing the results of the differences in the levels of mortality across different regions of the country.*

**2) The primary sample unit used did not match with the tertiary (i.e ITU bed) health care service provided in macro or microregion defined by Brazilian Health System (SUS). In other hand, there is available data regarding National health care survey (PNAS) about health services provided by microregion (or cities)...The analysis conducted did not support the conclusions.**

*Response: Pesquisa Nacional de Saúde (PNS) collected several health indicators, including information on comorbidities. Unfortunately, PNS does not have information for the microregion level. The sample size is 80,000 households and allows the estimation of some indicators for states, state capitals and metropolitan regions.*

*See the articles:*

*Szwarcwald, Celia Landmann, et al. "Pesquisa Nacional de Saúde no Brasil: concepção e metodologia de aplicação."* ***Ciência & Saúde Coletiva*** *19 (2014): 333-342.*

*Stopa, Sheila Rizzato, et al. "Use of and access to health services in Brazil, 2013 National Health Survey."* ***Revista de saude publica*** *51 (2017): 3s.*

*In addition, we included paragraphs in the analysis, to answer important and relevant issues raised by the reviewer, analysis based on previous work looking at the overall mortality levels across small-areas in Brazil and the spatial distribution of mortality by causes of deaths as an indicator of comorbidities and health conditions across areas. The analysis uses data from 2017 and 2018.*

*In relation to cardiovascular diseases, we observed that in approximately 75% of Brazilian micro-regions, men have higher mortality rates than women. The North and Northeast regions are the least developed socioeconomically regions of the country and have mortality rates bigger than the others. Previous studies show that less developed areas have observed the fastest rate of increase in mortality from cardiovascular diseases in recent years. Finally, diabetes mellitus affects mainly women. In approximately 78% of Brazilian micro-regions, mortality rates are higher for them compared to men. However, the spatial distribution suggests that, for males, this cause is more heterogeneous when analyzing the whole country. Spatial analysis also shows that, for both men and women, there is a concentration of high rates of mortality from diabetes in regions of the North and Northeast of the country. Additionally, the age structure is less aged in many microregions of the North and Northeast, on the other hand, in these regions it is where the highest overall mortality rates are presented when we control for population age structure. We also found that these areas, even before the pandemic, presented greater risks of general mortality and did not have a good health infrastructure (CODEÇO et al., 2020; SOUZA et al., 2020).*

*To emphasize our argument, we also include a discussion from a recent paper by Borges and Crespo (2020) using PNS data from 2013. They show, for the country, that age is the principal risk factor for comorbidities associated with COVID-19, but sociodemographic variables also have important impacts, generally indicating higher risk for persons in more vulnerable categories, such as those with less schooling and blacks and browns. They also show the prevalence of certain diseases such as diabetes and high blood pressure across different age groups and other SES categories.*

*Borges, G. M., & Crespo, C. D. (2020). Demographic and socioeconomic characteristics of Brazilian adults and COVID-19: a risk group analysis based on the Brazilian National Health Survey, 2013.* ***Cadernos de saude publica****, 36, e00141020.*

**3) The authors did not adjust by co-morbidities, however the Brazilian MoH conducted national surveys about non communicable diseases, with data available to make adjust about these diseases.**

*Response: on the issue of comorbidity, our analysis aggregate data for each of the municipality of Brazil and thus, the unit of analysis is the municipalities not individuals. We are of the view that data on comorbidity would be individual based and so, it would not be appropriate to include them in the present study.*
